# Evaluation of Free Radical Scavenging Activity of an Ayurvedic Formulation, *Panchvalkala*

**DOI:** 10.4103/0250-474X.40328

**Published:** 2008

**Authors:** Sheetal Anandjiwala, M. S. Bagul, M. Parabia, M. Rajani

**Affiliations:** Pharmacognosy and Phytochemistry Department, B. V. Patel Pharmaceutical Education Research Development (PERD) Centre, Thaltej, Ahemdabad - 380 054, India; 1 Department of Biosciences, South Gujarat University, Surat - 395 007, India

**Keywords:** Antioxidant, DPPH, superoxide radicals, reducing power assay, *Panchvalkala*

## Abstract

We report the free radical scavenging activity of an Ayurvedic preparation *Panchvalkala* and its individual components (stem bark of *Ficus benghalensis*, *F. glomerata*, *F. religiosa*, *F. virens* and *Thespesia populnea*). Being stem barks, these samples contain phenolics (ranging from 3.5% to 10.8% w/w) and tannins (1.6% to 7.0% w/w). This prompted us to study the free radical scavenging activity of *Panchvalkala* and its components which was evaluated in three *in vitro* models *viz*. 1,1-diphenyl-2-picrylhydrazyl radical scavenging activity, superoxide radical scavenging activity and reducing power assay. *Panchvalkala* and its individual components showed significant antiradical activity by bleaching 1,1-diphenyl-2-picrylhydrazyl radical (EC_50_ ranging from 7.27 to 12.08 µg) which was comparable to pyrogallol (EC_50_ 4.85 µg). Thin layer chromatography of the methanol extracts when sprayed with 0.2% 1,1-diphenyl-2-picrylhydrazyl in methanol revealed several bands with antiradical activity as seen by bleaching of 1,1-diphenyl-2-picrylhydrazyl. All the samples showed good superoxide scavenging potential (EC_50_ ranging from 41.55 to 73.56 µg) comparable to ascorbic acid (EC_50_ 45.39 µg) in a dose-dependent manner. The reduction ability, Fe^3+^ to Fe^2+^ transformation was found to increase with increasing concentrations of all the sample extracts.

Normally free radicals of different forms are generated at a low level in cells to help in the modulation of several physiological functions and are quenched by an integrated antioxidant system in the body. However, if produced in excess amount they can be destructive leading to inflammation, ischemia, lung damage and other degenerative diseases^1^. Many plant extracts and phytochemicals, especially the phenolic compounds such as flavonoids and benzyl-isothiocynate, have been shown to have antioxidant/free radical scavenging properties^2^.

*Panchvalkala* a reputed Ayurvedic preparation, is an equally proportioned mixture of dry powder of stem bark of 5 plants, *Ficus benghalensis*, *F. glomerata*, *F. religiosa*, *F. virens* and *Thespesia populnea*. It is astringent in taste, coolant, cures burning and quenches thirst^3^. The decoction is extensively used as antiinflammatory, to clear ulcers, dress wounds, as a douche in leucorrhoea and other vaginal diseases^4^. It is also used for oral consumption as a gargle in salivation. In scabies affected children, the decoction of *Panchvalkala* is administered externally and internally. *Panchvalkala* also forms a part of certain formulations for diarrhea and leucorrhoea^4^. We report our work on the free radical scavenging activity of *Panchvalkala* and its individual components.

## MATERIALS AND METHODS

Stem bark of the 5 plants, *Ficus benghalensis* (FB), *F. glomerata* (FG), *F. religiosa* (FR), *F. virens* (FV) and *Thespesia populnea* (TP) were collected from Ahmedabad, Gujarat. The samples were authenticated by our taxonomist and voucher specimens were preserved in the Pharmacognosy and Phytochemistry department. Plant materials were dried and stored in air tight containers and were powdered to 40 mesh as and when required. *Panchvalkala* (PV) was prepared by mixing equal amounts of powders (by weight) of the above five barks.

Ethylene diamine tetra acetate (EDTA) and Folin Ciocalteu's reagent were purchased from SD Fine Chemicals, Mumbai, India. 1,1-Diphenyl-2-picryl hydrazyl (DPPH), riboflavin, nitro blue tetrazolium chloride (NBT) and pyrogallol were purchased from Himedia Ltd, India. Potassium ferricyanide was purchased from Qualigens Fine Chemicals, India. Trichloroacetic acid (TCA) and Iron (III) chloride (FeCl_3_) from E. Merck India Ltd. Indigo Caramine was purchased from S. D. Fine Chemicals, India. Ascorbic acid was a gift sample from Cadila Pharmaceuticals Ltd., India. Gallic acid and tannic acid were gift samples from Tetrahedron Ltd., India. UV/Vis Spectrophotometer (Elico-India; SL-164) was used for spectrophotometric analysis.

### Preparation of methanol extract:

Ten grams each of the powders of *Panchvalkala* and its individual components were extracted separately with methanol (4 × 50 ml) under reflux at 70°. The extracts were filtered using Whatman 1 filter paper, pooled and concentrated to dryness under reduced pressure.

### Preliminary phytochemical testing:

Five hundred milligrams of the dried methanol extract was reconstituted in 10 ml of methanol and it was subjected to preliminary phytochemical testing for the presence of different chemical groups of compounds as per the methods previously reported by us^5^.

### Estimation of total phenolic content:

The total phenolic content of the extract was estimated according to the method described by Singleton and Rossi^6^. Briefly the method is as follows; Ten milligrams of standard gallic acid was dissolved in 100 ml distilled water in a volumetric flask (100 µg/ml of stock solution). From the above stock solution 0.5 to 2.5 ml of aliquots were pipetted out into 25 ml volumetric flasks. Ten ml of distilled water and 1.5 ml of Folin Ciocalteu's reagent (diluted according to the label specification) were added to each of the above volumetric flasks. After 5 min, 4 ml of 20% sodium carbonate solution was added and the volume was made up to 25 ml with distilled water and incubated at room temperature for 30 min and the absorbance of the solution was recorded at 765 nm and a standard curve of absorbance verses concentration of gallic acid (50-250 μg) was plotted.

One gram of the powdered drug was extracted with 70% methanol (15 × 3 times), filtered, pooled and the volume was adjusted to 50 ml with 70% methanol in a volumetric flask. From the stock solution, suitable quantity of the extract was taken into a 25 ml volumetric flask and 10 ml of water and 1.5 ml of Folin Ciocalteu reagent were added to it. The mixture was kept for 5 min, and then 4 ml of 20% sodium carbonate solution was added and made up to 25 ml with double distilled water. The mixture was incubated at room temperature for 30 min and the absorbance was recorded at 765 nm in a spectrophotometer. Percentage of total phenolics was calculated from calibration curve of gallic acid (50-250 µg) plotted using the above procedure and total phenolics were expressed as % gallic acid.

### Estimation of total tannins^7^:

Two grams of the powdered drug was extracted for 20 h with petroleum ether. The residue was boiled for 2 h with 300 ml of double distilled water. It was cooled, filtered with Whatman No. 1 filter paper and diluted to 500 ml with double distilled water. 25 ml of this infusion was pipetted into 2 litre porcelain dish to which 20 ml indigo solution and 750 ml double distilled water was added. This was titrated with standard KMnO_4_ (0.1 N) solution by adding 1 ml at a time, until blue solution changed to green, after which a few drops were added at a time until solution turned golden yellow in colour (A). Similarly, a mixture of 20 ml indigo solution and 750 ml of double distilled water was titrated (B). The percentage of total tannins was calculated using the formula, % Total tannins = [(A-B) × Actual Normality of KMnO_4_ solution × 0.004157 × 1000]/Weight of drug sample taken × 0.1. Each ml of 0.1 N KMnO_4_ ≅ 0.004157 g of total tannins.

### Free radical scavenging activity:

Hundred milligrams of dried methanol extract was dissolved in 100 ml of methanol to make a stock solution of 1 mg/ml. Aliquots from this stock solution were further diluted with methanol as per the concentrations required. Free radical scavenging activity of the methanol extract was tested in three *in vitro* models, *viz*., antiradical activity using DPPH^5^,^8^,^9^, superoxide radical scavenging activity in riboflavin-light-NBT system^5^,^10^ and reducing power assay by the transformation of Fe^3+^ to Fe^2+^ in the presence of the extracts^11^. The reaction mixtures for the assays are given below:

Antiradical activity was measured by a decrease in absorbance at 516 nm of a solution of coloured DPPH in methanol brought about by the sample^5^,^8^–^10^. A stock solution of DPPH (1.3 mg/ml in methanol) was prepared such that 75 µl of it in 3 ml methanol gave an initial absorbance of 0.9. Decrease in the absorbance in the presence of sample extract at different concentrations was noted after 15 min. EC_50_ was calculated from % inhibition. A blank reading was obtained using methanol instead of the extract. Pyrogallol was used as positive control^5^,^10^. Suitably diluted stock solution of methanol extracts of the stem bark were spotted on TLC plate and were developed in the solvent system of *n*-butanol:acetic acid:water:methanol:ethyl acetate (5:1:2:2:3). The plates were sprayed with 0.2% DPPH in methanol. Bleaching of DPPH by the resolved bands was observed for 10 min and the details were recorded.

Assay for superoxide radical scavenging activity was based on the capacity of the sample to inhibit blue formazan formation by scavenging the superoxide radicals generated in riboflavin-light-NBT system^5^,^10^,^12^. The reaction mixture contained 50 mM phosphate buffer (pH 7.6), 20 µg riboflavin, 12 mM EDTA, NBT 0.1 mg/3 ml, added in that sequence. The reaction was started by illuminating the reaction mixture with different concentrations of sample extract for 150 s. Immediately after illumination, the absorbance was measured at 590 nm and EC_50_ was calculated. Methanol was used for blank reading. Ascorbic acid was used as positive control^5^,^10^.

The reducing capability of the sample extracts was measured by the transformation of Fe^3+^ to Fe^2+^ in the presence of the extract. Increased absorbance of the reaction mixture indicates increased reducing power^11^. Different concentrations of extracts in 1 ml of water were mixed with 2.5 ml of phosphate buffer and 2.5 ml of potassium ferricyanide (1%). The mixture was incubated at 50° for 20 min, 2.5 ml of trichloroacetic acid (10%) was added to the mixture, centrifuged at 3000 rpm for 10 min, 2.5 ml of upper layer of the mixture was mixed with 2.5 ml distilled water and 0.5 ml of FeCl_3_ solution (0.1%) and the absorbance was measured at 700 nm. Increased absorbance of the reaction mixture indicated increased reducing power. Gallic acid and tannic acid were used as positive control.

## RESULTS AND DISCUSSION

In the living system, free radicals of different forms are constantly generated for specific metabolic requirement. When the generation of these species exceeds the levels of antioxidant mechanism, they cause extensive damage to the cells leading to oxidative damage of tissues and biomolecules, eventually leading to disease conditions, especially degenerative diseases and extensive lysis^13^. The living system is protected from this by enzymes such as superoxide dismutase, glutathione peroxidase and catalase and certain endogenous antioxidants such as α-tocopherol, ascorbic acid, β-carotene and uric acid^14^. Since the endogenous antioxidants acting as intracellular defense systems protecting cells from free radical damage and extensive lysis^15^, scavenging and diminishing the formation of oxygen-derived species are not 100% efficient, micro nutrients or antioxidants taken as supplements are particularly important in diminishing the cumulative oxidative damages^16^.

Various disease conditions are associated with free radical oxidative stress^12^. Herbal drugs containing free radical scavengers like phenolics, tannins and flavonoids are known for their therapeutic activity^5^,^8^,^10^,^13^. In the present study, preliminary phytochemical testing showed the presence of high amount of tannins and phenolics in all the samples (Table 1). Subsequent quantification revealed that the total phenolic content ranged from 3.5 to 10.8% w/w and the total tannin content ranged from 1.6 to 7.0% w/w in the samples (Table 2). The presence of high amount of phenolics and tannins and the above reasons prompted us to study the free radical scavenging activity of *Panchvalkala* and its individual ingredients.

**TABLE 1:** PRELIMINARY PHYTOCHEMICAL SCREENING OF *PANCHVALKALA* AND ITS INDIVIDUAL COMPONENTS

Chemical group	FB	FG	FR	FV	TP	PV
Phenols	++	+++	+++	++	+++	+++
Tannins	++	+++	+++	++	++	+++
Steroids/terpenoids	++	++	++	+	++	++
Alkaloids	+	+	+	+	++	+
Anthraquinones	-	-	-	-	-	-
Flavonoids	++	+++	++	++	+++	++

+++ means abundant;

++ denotes average;

- represents absent. FB, *Ficus benghalensis*; FG, *F. glomerata; FR, F. religiosa; FV, F. virens*; TP, *Thespesia populnea* and PV, *Panchvalkala*

**TABLE 2:** TOTAL PHENOLIC AND TOTAL TANNIN CONTENT OF *PANCHVALKALA* AND ITS INDIVIDUAL COMPONENTS

Sample	Total phenolics (% w/w)*	Total tannins (% w/w)*
*Ficus benghalensis*	03.59 ± 0.01	02.51 ± 0.01
*Ficus glomerata*	10.80 ± 0.23	07.03 ± 0.15
*Ficus religiosa*	07.89 ± 0.01	03.53 ± 0.07
*Ficus virens*	03.84 ± 0.03	01.64 ± 0.07
*Thespesia populnea*	10.11 ± 0.14	02.98 ± 0.01
*Panchvalkala*	06.89 ± 0.21	03.45 ± 0.15

* Mean % SD (*n* = 3)

**TABLE 3:** FREERADICAL SCAVENGING ACTIVITY OF METHANOL EXTRACT OF *PANCHVALKALA* AND ITS INDIVIDUAL COMPONENTS

Sample	Antiradical activity with DPPH EC_50_ (µg)	Superoxide radical scavenging activity EC_50_ (µg)
*Ficus benghalensis*	11.11	41.55
*Ficus glomerata*	07.59	66.91
*Ficus religiosa*	11.75	50.65
*Ficus virens*	07.27	59.82
*Thespesia populnea*	12.08	73.56
*Panchvalkala*	11.31	48.17
Pyrogallol	4.85	-
Ascorbic acid	-	45.39

**TABLE 4:** REDUCING POWER ASSAY OF METHANOL EXTRACTS OF *PANCHVALKALA* MEASURED BY THE TRANSFORMATION OF FE^3+^ TO FE^2+^

Sample	Concentration (µg)	Absorbance*
*Ficus benghalensis*	50	0.298 ± 0.007
	100	0.536 ± 0.005
	150	0.747 ± 0.006
	200	0.932 ± 0.025
	300	1.054 ± 0.014
*Ficus glomerata*	50	0.195 ± 0.011
	100	0.361 ± 0.006
	150	0.477 ± 0.003
	200	0.661 ± 0.001
	250	0.867 ± 0.010
	300	1.018 ± 0.013
*Ficus religiosa*	50	0.164 ± 0.028
	100	0.355 ± 0.015
	150	0.510 ± 0.002
	200	0.662 ± 0.008
	300	0.766 ± 0.035
	400	1.092 ± 0.001
*Ficus virens*	50	0.190 ± 0.013
	100	0.370 ± 0.036
	150	0.590 ± 0.004
	200	0.768 ± 0.004
	300	0.946 ± 0.018
	400	1.304 ± 0.006
*Thespesia populnea*	50	0.149 ± 0.005
	100	0.400 ± 0.002
	150	0.566 ± 0.013
	200	0.848 ± 0.004
	300	1.012 ± 0.003
*Panchvalkala*	50	0.244 ± 0.001
	100	0.363 ± 0.004
	150	0.555 ± 0.042
	200	0.775 ± 0.003
	300	1.178 ± 0.030
Gallic acid	5	0.088 ± 0.008
	10	0.183 ± 0.001
	20	0.523 ± 0.031
	50	1.218 ± 0.015
Tannic acid	5	0.146 ± 0.019
	10	0.306 ± 0.008
	20	0.710 ± 0.010
	50	1.482 ± 0.034

* Mean % SD (n = 3)

**Fig. 1:**
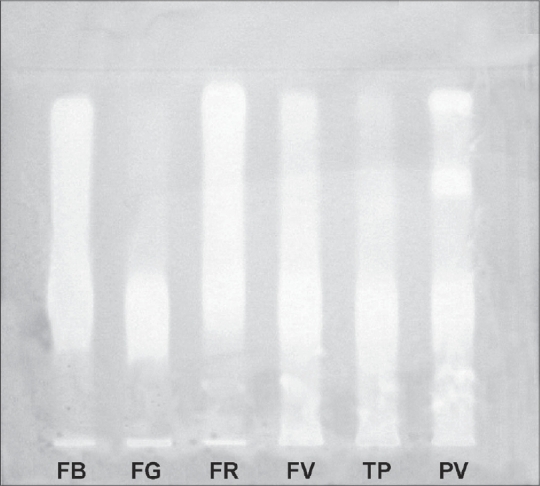
TLC of *Panchvalkala* and its ingredients sprayed with 0.2% methanol DPPH. FB, *Ficus benghalensis*; FG, F. *glomerata*; FR, F. *religiosa*; FV, F. *virens*; TP, *Thespesia populnea*; PV, *Panchvalkala*

Free radical scavenging action is considered to be one among the various mechanisms for antioxidation^17^. We studied antiradical activity of methanol extract of *Panchvalkala* and its individual components by testing its ability to bleach the stable DPPH radical. This method is based on the reduction of alcoholic DPPH solution in the presence of hydrogen donating antioxidant (AH) due to the formation of non-radical form DPPH-H by the reaction DPPH + AH → DPPH-H + A. The remaining DPPH measured after a certain time, corresponds inversely to the radical scavenging activity of the antioxidant^18^. The sensitivity of the method is determined by the strong absorption of DPPH. This method is rapid, a sample analysis takes only 15 min and little manpower, no expensive reagents or sophisticated instruments are required^18^. This assay is being used widely as a preliminary test which provides information on the reactivity of test compound with a stable free radical since odd electron of DPPH gives strong absorption band at 517 nm (violet colour) and when it is quenched by the extract, there is a decrease in absorbance. Methanol extract of *Panchvalkala* and its individual components showed a very good antiradical activity (FV = FG < FB = PV = FR < TP) in scavenging DPPH radical (comparable to the positive control, pyrogallol) with a maximum inhibition of about 85% (EC_50_ ranged from 7.27 µg to 12.08 µg for the samples analyzed) (Table 3). A TLC plate on which methanol extract was applied and developed in the solvent system of *n*-butanol:acetic acid:water:methanol:ethyl acetate (5:1:2:2:3) and sprayed with 0.2% DPPH in methanol showed bands that bleached DPPH. A streak of discolouration of DPPH was observed along the tracks of all the six samples due to bleaching of DPPH. In *Ficus benghalensis*, the track got bleached from R_f_ 0.25 to 0.88; in *F. glomerata*, the bands at R_f_ 0.70 and 0.86, and also from the application point to R_f_ 0.44 bleached DPPH, in *F. religiosa* the track bleached DPPH from R_f_ 0.29 to 0.91; in *F. virens* and *Thespesia populnea* the track bleached DPPH from the application point to the solvent front and in *Panchvalkala* the bands at R_f_ 0.14, 0.20, 0.29 to 0.44, 0.63 and 0.90 bleached DPPH (fig. 1).

The superoxide radical is ubiquitous in aerobic cells^19^. Although only mildly reactive toward biological molecules, the superoxide radical may be transformed to the highly reactive and damaging hydroxyl radical^20^. All the samples showed good superoxide scavenging potential (FB < PV < FR < FV < FG < TP) in a concentration-dependent manner (EC_50_ 41.55 µg to 73.56 µg in the six samples). The activity of *F. benghalensis* and *Panchvalkala* was found to be comparable to ascorbic acid which was used as positive control (Table 3).

Reducing power assay is a convenient and rapid screening method for measuring the antioxidant potential^11^. The reduction ability (“Fe^3+^ to Fe^2+^ transformation” in terms of increasing absorbance) was found to increase with rising concentration in all the samples. About 400 µg of methanol extract of all the samples were shown to have maximum reducing power (absorbance ~1.09), which was comparable to that of gallic acid and tannic acid (considering the amount of tannins and phenolics present in the samples) which were used as positive control which gave maximum absorbance at a concentration of 50 µg (Table 4).

From the above experiments it is clear that *Panchvalkala* and its components showed good free radical scavenging activity which can be attributed to tannins and phenolics along with other compounds. Free radical scavenging activity could be one of the mechanisms of action of *Panchvalkala*, including its anti-inflammatory activity.
